# The Association Between Perceived Discriminatory Climate in School and Student Performance in Math and Reading: A Cross-National Analysis Using PISA 2018

**DOI:** 10.1007/s10964-022-01712-3

**Published:** 2022-12-07

**Authors:** Gülseli Baysu, Orhan Agirdag, Jozefien De Leersnyder

**Affiliations:** 1 Queen’s University of Belfast, Belfast, UK; 2 University of Leuven, Leuven, Belgium

**Keywords:** Discrimination, School climate, PISA 2018, Achievement, Ethnic minority

## Abstract

The negative consequences of perceived ethnic discrimination on adolescent adjustment are well documented. Less is known, however, about the consequences of discriminatory *climates* in school, beyond the individual experiences of discrimination. This study investigated whether a perceived discriminatory climate in school is associated with lower academic performance across adolescents from ethnic minority and majority groups, and which psychological mechanisms may account for this link. Using the 2018 Programme for International Student Assessment (PISA) data, the participants were 445,534 adolescents (aged 15–16, 50% girls) in 16,002 schools across 60 countries. In almost all countries, a discriminatory climate*—*i.e., student perceptions of teachers’ discriminatory beliefs and behaviors in school*—*was associated with lower math and reading scores across all pupils, although minorities perceived a more discriminatory climate. Lower school belonging and lower values attributed to learning partially mediated these associations. The findings demonstrate that schools’ ethnic and racial climates predict standardized academic performance across schools and countries among pupils from both ethnic majority and minority groups.

## Introduction

A large body of empirical research documents how academic outcomes of adolescents from ethnic minority groups are negatively impacted by experiences of discrimination (Benner et al. 2018). As adolescence is the period when young people’s views on equality and diversity are formed, they can become more aware of and more susceptible to discrimination or unfairness during this time (Baysu et al. 2016). There is also increasing evidence to suggest that merely witnessing discrimination, even without being the target, may jeopardize one’s sense of belonging and engagement (Jaurique et al. 2018). These findings imply that adolescents do not need to experience discrimination themselves to be negatively impacted by the discrimination they perceive in their school environment. To date, however, not much is known about the consequences of discriminatory *climates* in schools beyond the individual experiences of discrimination (Benner, 2017). Moreover, although this topic is internationally relevant, the research on ethnic discrimination is heavily weighted toward US samples (Benner et al. 2018), and a cross-national analysis providing robust evidence for its negative impact is lacking. Addressing these research gaps, the current study aimed to examine the associations between the perceived discriminatory climates in schools and academic performance among pupils from ethnic minority and majority groups across thousands of schools and 60 countries using the 2018 Programme for International Student Assessment (PISA) data. The measure of *perceived discriminatory climate* focused on pupils’ perceptions of discriminatory beliefs and behaviors among teachers as representatives of the school. In addition, this study aimed to shed some light on which psychological mechanisms, such as pupils’ sense of belonging to the school and the value they attribute to learning, may account for the link between discriminatory school climates and academic achievement. A further objective was to document the differential levels and effects of discriminatory climates among pupils from ethnic majority vs. minority groups via both mediation and moderation tests.

### Discriminatory Climate in School and Academic Achievement

To explain how a discriminatory climate in school is associated with adolescents’ academic achievement, this study takes an ecological perspective (Bronfenbrenner and Morris, 2006) and draws on two lines of social psychological and educational research that have developed quite separately: research on school climates (Wang and Degol, 2016) and studies on ethnic discrimination (Umaña-Taylor, 2016). Ecological theory articulates how development should be situated in multi-layered contexts. Accordingly, schools form one of the critical levels of micro-contexts that can influence adolescent adjustment through proximal processes, referring to the complex interactions between the adolescent and their contextual environments like schools (Bronfenbrenner and Morris, 2006). The most widely studied construct that captures the school environment is *school climate*. While school climate is considered to be a complex and multi-dimensional construct (Thapa et al. 2013), most research conceptualizes it in terms of shared perceptions of school norms, values, and expectations, as well as interpersonal and intergroup interactions (Wang and Degol, 2016). For instance, school climate can refer to how welcomed, valued, and respected pupils feel (UNESCO, 2022), which are features that affect student learning and school functioning (Thapa et al. 2013).

Given the high ethnic, racial, and religious diversity in today’s classrooms, more and more research, including the present study, has focused on schools’ multicultural or racial/ethnic school climates, which is often referred to as a school’s *diversity climate* (see, e.g., Chang and Lee, 2010; Baysu et al. 2021). There is generally a consensus that students’ perceptions of a positive school diversity climate contribute to their academic outcomes, such as their academic achievement and adjustment levels (see, e.g., Chang and Le, 2010 for an empirical study, and Wang and Degol, 2016 for a review), even after controlling for other factors that have been shown to predict academic outcomes, such as socioeconomic status (SES), school track, and school composition (Schachner et al. 2019). Nevertheless, it has also been found that students from ethnic minority groups often experience a less positive school diversity climate than their peers from ethnic majority groups (Thapa et al. 2013), which may translate into lower school achievement. Beyond the individual perceptions of the diversity climate (Benner and Graham, 2011), recent studies have increasingly focused on shared/aggregated/collective perceptions of schools’ diversity climates. For instance, studies focused on the students’ and teachers’ school-level shared perceptions (Baysu et al. 2021), students’ classroom-level shared perceptions (Schachner et al. 2019), or actual school-level policies and practices favoring different diversity approaches (Celeste et al. 2019). Doing so has revealed that students from ethnic minority groups are more likely to suffer from a school diversity climate that disvalues their identities by either ignoring them or pressuring them to assimilate (Celeste et al. 2019), or one that attaches low intrinsic moral value to diversity (Starck et al. 2021). In contrast, pupils from ethnic minority groups benefit from positive diversity climates that value their identities (e.g., Celeste et al. 2019)—a benefit that is increasingly found among pupils from ethnic majority groups as well and that manifests itself as higher-quality teacher-student relationships (Baysu et al. 2021), higher achievement, and fewer disciplinary problems (Mattison and Aber, 2007), as well as higher school belonging (Schachner et al. 2019). In sum, this line of research has shown that schools’ perceived diversity climates matter to the academic outcomes of pupils from both minority and majority groups.

While these studies on school diversity climates have focused on various aspects of diversity, ranging from cultural appreciation (Chang and Le, 2010) to intergroup contact opportunities (Schachner et al. 2019) and efforts in combating discrimination (Baysu et al. 2021) and increasing fairness (Mattison and Aber, 2007), almost no studies have focused on pupils’ perceptions of a *discriminatory climate* (i.e., aggregated and individual perceptions of discriminatory treatment toward particular demographic groups by their organization). This is a missed opportunity since there is much evidence that experiencing discrimination puts children at risk of poorer developmental outcomes (Syed et al. 2018). Specifically, ethnic minority adolescents’ *own* experiences of ethnic discrimination have been systematically associated with worse academic outcomes at the individual level (Benner et al. 2018) across all genders (Benner et al. 2018), even after controlling for contextual variables, such as school track and composition (Baysu et al. 2016). Moreover, ethnic minorities often experience more discrimination than ethnic majority groups (e.g., Verkuyten et al. 2019). When students from ethnic minority groups perceive ethnic discrimination from teachers as compared to peers (Benner and Graham, 2013), they show reduced school belonging (Brown and Chu, 2012), lower academic engagement (Verkuyten and Thijs, 2004), and lower academic performance (Benner and Graham, 2013).

In addition, there is increasing evidence that merely witnessing discrimination—sometimes called indirect (Huynh et al. 2017), vicarious (Alvarez et al. 2006), or ambient discrimination (Chrobot-Mason et al. 2013)—may also jeopardize one’s sense of belonging and school engagement (Jaurique et al. 2018). While students from minority groups witnessing discrimination also report higher levels of exhaustion (Harwood et al. 2012), those who witness discrimination without being the target are impacted as well. For instance, one study showed that the more university students witnessed examples of discrimination toward their peers, the lower their university identification and academic engagement, and the higher their levels of anxiety and depression were (Smith et al. 2016). Generally, people report high frequencies of witnessing discrimination in educational settings. For instance, among young adolescents in Australia, 22.1% experienced racism each day, and 47.3% observed it (Priest et al. 2014). Therefore, the potential negative effects of perceived school discriminatory climates cannot be overestimated. Moreover, discrimination in a given context, such as school, could be covert and subtle, and thus invisible to some individuals, particularly those less victimized by it. Other pupils in the same school could be more aware of it and report it as such. The fact that discrimination is less visible for some individuals does not mean that it is less harmful, even for those who perceive it less. Hence, it is critical to examine both personally perceived discriminatory climates at the individual level and shared perceptions of discriminatory climates at the school level.

In light of these developments, this study sought to respond to a recent review on racial and ethnic discrimination that concluded that there is a “need for greater attention to school climate in research” (Benner, 2017, p. 254), because even when individual students do not experience ethnic discrimination themselves, experiencing a discriminatory climate might have adverse consequences (see also the review of Jaurique et al. 2018). Therefore, the present research examines the link between pupils’ individual and shared perceptions of the *discriminatory school climate* (i.e., their teachers’ discriminatory behavior toward specific ethnic minority groups) and their standardized academic achievement. Specifically, it was hypothesized (*Hypothesis 1*) that perceived discriminatory climate in school is associated with lower math and reading scores at both the individual and school levels across the 60 countries in the PISA dataset. Using the PISA dataset has several advantages. First, it focuses on math and reading scores, as these reflect crucial outcomes for individuals in society. Mathematical literacy is regarded as an individual’s competency to formulate, employ, and interpret math to solve problems in real-world contexts. Reading literacy is regarded as an individual’s capacity to understand, evaluate, reflect on, and engage with texts to achieve one’s goals, develop one’s knowledge and potential, and participate in society. Second, the PISA data focus on 15-year-olds, which corresponds to middle adolescence. A meta-analysis of discrimination (Benner et al. 2018) revealed that the impact of discrimination on the academic domain was the largest in middle adolescence compared to early or late adolescence. Furthermore, most adolescents at the age of 15 are still enrolled in formal education in most countries. Thus, middle adolescence constitutes a good period for studying the association between discriminatory climates in school and academic achievement. Thirdly, the 2018 PISA dataset includes data from 60 countries, which allows for testing the robustness of the effects across many different countries. Finally, to more reliably show the unique associations between performance and perceived discriminatory school climates, the potential confounding effects of certain variables were also considered. Specifically, gender (Agirdag and Vanlaar, 2018), a student’s educational track (Baysu et al. 2018) and SES (APA, 2020) at the individual level, and the percentage of students from ethnic minority and low SES backgrounds at the school level (Rjosk et al. 2017) can reliably predict achievement; thus, these were included as control variables.

Furthermore, the current research investigates the differential levels and effects of perceived discriminatory climate on the achievement of pupils from both ethnic minority and majority groups—something that both lines of research on discrimination and school diversity climates have only started to do recently. Following Mattison and Aber (2007), the interplay between ethnic minority status and the link between discriminatory school climate and achievement can be examined in two ways. First, and based on studies that show more harm to the well-being of students from minority groups (Harwood et al. 2012), it could be expected that the negative association between discriminatory school climate and academic performance would depend on minority/majority group membership and would be more consequential (i.e., the effect would be larger) for students from ethnic minority groups than those from majority groups (*Hypothesis 2a*). Technically, this would be a *moderation hypothesis* that tests the interaction between ethnic minority status as a moderator and discriminatory school climate as a predictor and its effect on performance as an outcome. Alternatively, and based on studies that suggest that majority groups are less aware of ethnic discrimination than minorities (Tropp and Barlow, 2018), one could expect that students from ethnic minority groups would perceive more discrimination at school and thus report higher levels of discriminatory school climate, which would then predict lower academic performance (*Hypothesis 2b*). Technically, this would be a *mediation hypothesis* that tests for the negative indirect effect of minority status as a predictor via discriminatory school climate as a mediator of academic performance as an outcome. In other words, this suggests that minorities’ underachievement might be partially explained by their more frequent experiences of discriminatory climates. Thus, to fully examine the role of minority/majority group membership, both the mediation and moderation hypotheses were examined.

### School Belonging and Attitudes to Learning as Mediators Between the Discriminatory Climate in School and Achievement

To further understand *why* discriminatory school climates may be associated with lower achievement, two potential psychological mechanisms, namely “belonging” and “attitudes toward learning,” were examined. A positive school diversity climate can create a safe environment where students feel that they are respected and valued (Phalet and Baysu, 2020), and in such a context, embracing diversity and preventing discrimination have equal intrinsic moral value (Starck et al. 2021). On the contrary, experiencing (Major and O-Brian, 2005) or witnessing discrimination (Jaurique et al. 2018) are conceptualized as stressors that can lead to a heightened threat to the identity of the in-group, whether it is an ethnic identity (Verkuyten et al. 2019) or an organizational identity (Smith et al. 2016), as well as a heightened threat to morality (Jaurique et al. 2018) and fairness (Killen et al. 2013). Because discrimination can threaten one’s group identity and moral orientation, as a coping mechanism, people may respond by distancing themselves from the organization behaviorally or psychologically (for more on witnessing discrimination, see Jaurique et al. 2018; for experiencing discrimination, see Verkuyten et al. 2019). In other words, they can withdraw their efforts and disengage from (Smith et al. 2016) or attach a lower value to the domains that induce these threats (Major and O-Brian, 2005). Translating this research to the potential effects of discriminatory climates in school, these findings imply that a perceived discriminatory climate can be associated with reduced feelings of belonging to school and lower value attributed to learning and effort, and, in turn, with lower achievement. Moreover, from a developmental intergroup perspective (Killen et al. 2013), both identity-related and fairness-related concerns are salient during adolescence. It is thus critical to study these processes in adolescence.

While the feeling of belonging is a psychological need for all humans (Ryan and Deci, 2000), school belonging refers to the extent to which adolescents feel accepted, included, and supported in school (Schachner et al. 2019). It is considered an affective dimension of school engagement (Fredricks et al. 2004). School engagement in general, or school belonging as its affective component, has been found to predict higher achievement (Fredricks et al. 2004). Moreover, a student’s own experiences of discrimination (Baysu et al. 2016), witnessing discrimination (Smith et al. 2016), and perceptions of less positive school diversity climates (Schachner et al. 2019) have been associated with reduced belonging and, in turn, lower achievement; thus, the mediating role of school belonging has been established before (see Baysu et al. 2016 for discrimination, and Schachner et al. 2019 for school diversity climate). While school belonging is sometimes regarded as part of the school climate, such that certain schools can be defined as having a higher or lower quality of connectedness (Wang and Degol, 2016), this study focuses on students’ *own* experiences of belonging in school as a mediator between perceived discriminatory climate and academic performance.

In addition, attaching lower value to learning or devaluing the academic domain has been identified as another psychological coping mechanism for discrimination (Levy et al. 2016). Discrimination can lead to disillusionment about the real value of schooling and can thus discourage ethnic minorities from working hard in school (Levy et al. 2016). In other words, in the face of discrimination, while devaluing allows students to remove their self-worth from an academic domain, it also undermines their motivation to perform well in that domain (Verkuyten and Thijs, 2004). Attitudes toward learning activities, or specifically the value attributed to learning and studying, can also be likened to the concept of academic futility, which refers to the sense that the student has little or no control over their educational success and failures. For instance, research has shown that ethnic discrimination by teachers predicts an increased sense of academic futility among students from ethnic minority groups (D’hondt et al. 2016). Moreover, teachers’ expressions of low teachability expectations regarding pupils’ language use and alleged linguistic deficiencies were found to predict an increased sense of futility and, in turn, lower achievement among all students (Agirdag et al. 2013). These findings imply that the value attributed to learning can act as a mediator between discriminatory climates and academic performance.

Combining these two research lines, two tentative expectations were put forward (*Hypothesis 3*): When students perceived a more discriminatory climate in school, they would report lower school belonging (*Hypothesis 3a)* and more negative attitudes toward learning (*Hypothesis 3b*) and, consequently, perform worse in math and reading.

## Present Study

Less is known about the consequences of discriminatory *climates* in schools beyond the individual experiences of discrimination. Based on separate lines of research on experiencing and witnessing discrimination and on school diversity climates, (i) a negative association between the perceived discriminatory climate in school and students’ performance was expected (Hypothesis 1); (ii) the interplay of this link with ethnic minority status was explored via either moderation (Hypothesis 2a) or mediation (Hypothesis 2b); and (iii) school belonging and attitudes toward learning activities were tested as potential mediators between discriminatory school climate and achievement (Hypotheses 3a and 3b, respectively). To show the unique associations between performance and perceived discriminatory school climates more reliably, gender, educational track, and SES at the individual level and the percentage of students from ethnic minority and low SES backgrounds at the school level were included as control variables.

## Methods

### Participants

The sample was drawn from a large-scale international dataset called the Organisation for Economic Co-operation and Development (OECD) Programme for International Student Assessment (PISA) 2018. PISA examines student performance in reading and mathematics and is one of the most comprehensive international assessments of student learning outcomes. Following consent being obtained from the respective parties in line with the ethical guidelines, students participated in PISA 2018 during class hours in the presence of test administrators (OECD, 2019b). Computer-based tests are used in most countries, with assessments lasting a total of 2 h. This study focused on 445,534 adolescents in 60 countries that participated in PISA 2018. Their ages ranged from 15 to 16 years (*M* = 15.81, SD = 0.29). Half identified as female, and the other half as male. The majority (86%) followed a general track in school vs. vocational tracks (for further details on data collection, survey format, and access to datasets, see OECD, 2019a, 2019b).

### Measures

Measures were standardized for OECD countries in line with PISA’s scaling of indices; thus, the scores were transformed to have a mean of 0 and a standard deviation of 1 across OECD countries, except for the math and reading scores (for details on the scales, see the technical report in OECD, 2019b). Descriptive statistics for all study variables can be found in Table 1.

**Table 1:** Descriptive statistics of the main study variables

Variables	1	2	3	4	5	6	7	8	9	10	11	S1	S2	S3
1 Minority (vs. majority)														
2 Discrim. school climate	0.109													
3 Math	−0.189	−0.253												
4 Reading	−0.256	−0.327	0.839											
5 Attitudes to Learning	0.014	−0.089	0.004	0.050										
6 School belonging	−0.031	−0.137	0.125	0.151	0.185									
7 SES	−0.212	−0.079	0.477	0.475	−0.002	0.100								
8 Gender	−0.014	−0.136	−0.018	0.112	0.075	0.011	−0.026							
9 Track	0.042	−0.025	0.039	0.043	−0.021	−0.011	0.070	0.037						
10 Supportive climate	0.018	−0.084	−0.074	−0.048	0.141	0.119	−0.075	0.015	0.004					
11 Teacher support	0.038	−0.083	0.011	0.023	0.112	0.154	−0.051	0.038	−0.003	0.297				
School level														
S1 Discrim. school climate	0.230	0.406	−0.389	−0.427	−0.026	−0.129	−0.235	−0.070	−0.048	−0.003	−0.037			
S2 Ethnic composition	0.513	0.103	−0.261	−0.311	0.054	−0.015	−0.260	0.006	0.042	0.048	0.073	0.233		
S3 SES composition	0.176	0.101	−0.392	−0.375	0.064	−0.044	−0.406	−0.013	−0.082	0.060	0.053	0.242	0.288	
M	0.264	0.152	421.878	423.021	0.021	−0.140	−0.910	0.503	0.859	0.300	0.091	0.166	29.161	33.866
SD	0.441	1.036	100.774	103.316	1.017	0.903	1.260	0.500	0.349	0.926	0.939	0.439	39.102	30.063
Min 0	0	−1.16	24.74	21.86	−2.54	−3.85	−8.17	0	0	−2.74	−2.29	−1.15	0	0
Max	1	−3.18	863.08	887.69	1.08	3.98	4.00	1	1	1.34	1.55	3.18	100	100

Discrim. is short for discriminatory

All correlations significant at *p* < 0.001

#### Discriminatory school climate

It was measured with four items: “Thinking about teachers in your school: to how many of them do the following statements apply?” (1) “They have misconceptions about the history of some cultural groups,” (2) “They say negative things about people of some cultural groups,” (3) “They blame people of some cultural groups for problems faced by <country of test>,” (4) “They have lower academic expectations for students of some cultural groups” (α = 0.86; OECD, 2019b, Chapter 16). Answers ranged from 1 (*to none or almost none of them*) to 4 (*to all or almost all of them*; see Supplementary Online Materials Section 1, SOM.1, for Ms and SDs for each country). In addition to the individual-level standardized measure, the school-level aggregated student perceptions of discriminatory school climate were calculated.

#### The ethnic minority vs. majority group

This variable was defined (1 = *ethnic minority*; 0 = *ethnic majority*) based on immigration status (first generation, second generation, or non-immigrant) and language spoken at home (do/do not speak any other language at home). Adolescents from ethnic minority groups (26.4%) were either the first or second generation of an immigration background *or* spoke another language at home. Adolescents from ethnic majority groups (73.6%) identified as non-immigrant *and* did not speak any other language at home.

#### Academic performance

It was measured by students’ math (range: 25–863, *M* = 422) and reading performance scores (range: 22–888, *M* = 423) based on the PISA achievement tests that consist of multiple-choice items and short essay questions. The original PISA data contained 10 plausible values for each domain. The first plausible values of mathematics and reading scores were used in this study (for more information on PISA tests, see Mo, 2019).

#### School belonging

It was measured with six items: (1) “I feel like an outsider (or left out of things) at school,” (2) “I make friends easily at school,” (3) “I feel like I belong at school,” (4) “I feel awkward and out of place in my school,” (5) “Other students seem to like me,” and (6) “I feel lonely at school” (α = 0.80), each rated from 1 (*strongly disagree*) to 4 (*strongly agree*).

#### Attitudes toward learning activities

This variable was measured with three items: (1) “Trying hard at school will help me get a good job,” (2) “Trying hard at school will help me get into a good <college>,” and (3) “Trying hard at school is important” (α = 0.87), with each rated from 1 (*strongly disagree*) to 4 (*strongly agree*).

#### Control variables

Educational track (1 = general track; 0 = vocational track), socioeconomic status (SES; an index of highest parental education and occupation as well as household possessions), and gender (1 = girls; 0 = boys) were controlled at the individual level. At the school level, the percentage of students from ethnic minority and low SES backgrounds in school was controlled for.

To rule out the alternative explanation that the effects of discriminatory climate (as it is operationalized through teachers’ behaviors) are not specific to ethnic and racial issues but just the absence of general teacher support, additional robustness analyses were run with two variables about teacher support (i.e., supportive climate and perceived teacher support) during their language classes (e.g., English lessons in the UK). Both scales were rated from 1 (*never or hardly ever*) to 4 (*every lesson*).

#### Supportive climate

It was measured with four items: “During their <test language lessons>,” (1) “The teacher shows an interest in every student’s learning,” (2) “The teacher gives extra help when students need it,” (3) “The teacher helps students with their learning,” and (4) “The teacher continues teaching until the students understand” (α = 0.86).

#### Perceived teacher support

Teacher support was measured with three items: “Thinking of your past two <test language lessons>,” (1) “The teacher made me feel confident in my ability to do well in the course,” (2) “The teacher listened to my view on how to do things,” and (3) “I felt that my teacher understood me” (α = 0.86).

### Analytic Strategy

To test Hypothesis 1 on the effects of a discriminatory climate on pupils’ math and reading achievement, as well as the potential moderation by pupils’ minority status (Hypothesis 2a), multilevel regression analyses (three levels: students, schools, and countries) were run separately for math (Table 2) and reading scores (Table 3). This was done in a stepwise fashion with the *null model* (the random intercept model), *control-only model* (predictors at the individual level: SES, gender, track, ethnic minority; at the school level: ethnic and SES composition of schools), and *main effects model* (+ discriminatory school climate at the individual and school levels). Subsequently, ethnic minority background was tested as a moderator of discriminatory school climate effects in fixed-effects models (*moderation minority)* and random slope models, where the main effects of all the predictors were allowed to vary across the school and country levels (*random slopes*). Intraclass correlations (ICC) from the null model (Tables 2, 3) indicated that there was a large variation at the school and country levels in math and reading scores, justifying the choice of multilevel analyses.

**Table 2:** Stepwise multilevel regression models predicting PISA 2018 math scores

	Null model	Control only		Main effects		Moderation minority		Random slopes	
	Variance	*B*	SE	*B*	SE	*B*	SE	*B*	SE
*Ind level*									
SES		14.26^***^	1.354	14.44^***^	1.31	15.11^***^	1.33	15.44^***^	1.00
Gender		−9.53^***^	1.084	−12.36^***^	1.03	−13.68^***^	1.21	−14.66^***^	1.08
Track		23.85^***^	6.962	21.00^***^	6.28	21.57^***^	5.51	20.40^***^	4.85
Minority		−10.71^***^	2.406	−9.52^***^	2.31	−7.33^**^	2.39	−9.44^***^	1.77
Discrimination				−12.03^***^	0.62	−11.89^***^	0.57	−12.23^***^	0.57
Minority × Discrim						−2.35^**^	0.87	−0.61	0.43
R2		0.10^***^		0.10^***^		0.10^***^			
(Residual) variance	5312.60^***^	5146.01^***^		5011.23^***^		5006.39^***^		4818.87^***^	
*School level*									
Ethnic%		−0.04	0.03	−0.03	0.03	−0.02	0.03	−0.02	0.03
SES%		−0.70^***^	0.05	−0.57^***^	0.04	−0.53^***^	0.04	−0.52^***^	0.04
Discrimination				−37.16^***^	2.69	−39.48^***^	2.67	−37.05^***^	2.63
Random slopes variances									
SES								34.34^***^	
Gender								69.40^***^	
Track								540.28^***^	
Minority								217.13^***^	
Discrimination								12.72^***^	
Minority × Discrim								30.58	
R2		0.19^***^		0.31^***^		0.33^***^			
(Residual) variance	2812.04^***^	1557.00^***^		1241.82^***^		1128.64^***^		1111.93^***^	
*Country level*									
Mean (Math)	431.54^***^	460.22^***^		466.88^***^		469.99^***^		468.59^***^	
Variance (Math)	5141.38	1913.15^***^		1594.60^***^		1476.97^***^		1455.71^***^	
Random slopes variances									
SES								42.22^***^	
Gender								61.82^***^	
Track								1022.49^***^	
Minority								161.22^***^	
Discrimination								17.01^***^	
Minority × Discrim								2.32	

The table presents unstandardized regression coefficients and Standard Errors (SE). Intraclass correlations based on the null model at the school level 0.21, at the country level 0.39. Discrimination and discrim are short for discriminatory school climate

**p < 0.01; ***p < 0.001

**Table 3:** Stepwise multilevel regression models predicting PISA 2018 reading scores

	Null model	Control only		Main effects		Moderation minority		Random slopes	
	Variance	*B*	SE	*B*	SE	*B*	SE	*B*	SE
*Ind level*									
SES		14.11^***^	1.19	14.40^***^	1.12	14.92^***^	1.11	15.03^***^	0.94
Gender		21.23^***^	0.90	17.25^***^	0.81	16.43^***^	0.81	16.15^***^	0.96
Track		24.03^***^	7.11	20.53^***^	6.22	21.37^***^	5.54	21.97^***^	5.03
Minority		−15.01^***^	2.15	−13.33^***^	2.00	−11.11^***^	2.00	−14.10^***^	2.02
Discrimination				−17.46^***^	0.87	−17.26^***^	0.83	−17.43^***^	0.75
Minority × Discrim						−2.02^*^	0.93	−0.70	0.44
R2		0.08^***^		0.13^***^		0.13^***^			
(Residual) variance	5747.27^***^	5488.25^***^		5202.44^***^		5150.33^***^		4942.10^***^	
*School level*									
Ethnic%		−0.07	0.04	−0.05	0.04	−0.04	0.03	−0.03	0.03
SES%		−0.74^***^	0.05	−0.59^***^	0.04	−0.55^***^	0.04	−0.53^***^	0.04
Discrimination				−41.74^***^	2.54	−44.04^***^	2.73	−40.85^***^	2.57
Random slopes variances									
SES								36.48^***^	
Gender								83.28^***^	
Track								748.68^***^	
Minority								264.01^***^	
Discrimination								18.04^***^	
Minority × Discrim								33.96	
R2		0.20^***^		0.35^***^		0.37^***^			
(Residual) variance	3624.90^***^	1692.02^***^		1252.12^***^		1128.89^***^		1068.86^***^	
*Country level*									
Mean (Reading)	444.70^***^	443.36^***^		451.78^***^		454.76^***^		451.88^***^	
Variance (Reading)	5348.89^***^	1554.45^***^		1153.37^***^		1049.18^***^		1310.76^***^	
Random slopes variances									
SES								35.88^***^	
Gender								43.19^***^	
Track								1052.40^***^	
Minority								222.82^***^	
Discrimination								28.97^***^	
Minority × Discrim								2.21	

The table presents unstandardized regression coefficients and Standard Errors (SE). Intraclass correlation based on the null model at the school level 0.25, at the country level.36. Discrimination and discrim are short for discriminatory school climate

*p < 0.05; ***p < 0.001

**Fig. 1:**
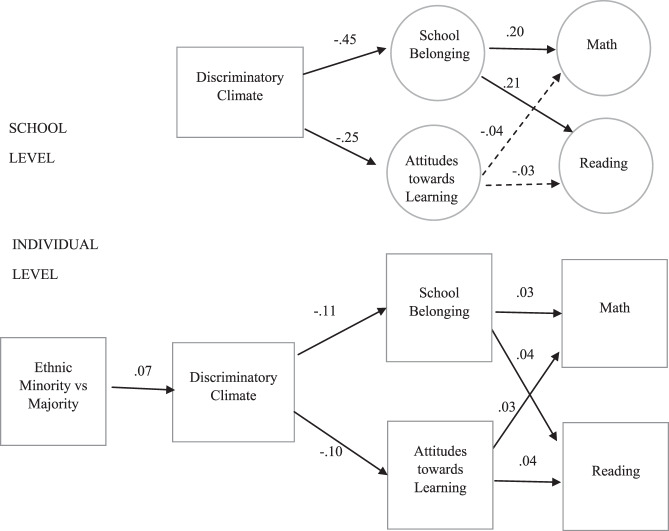
Multilevel mediation model predicting PISA 2018 Math and Reading Scores. The figure presents the standardized regression coefficients (SDYX) from two separate models for math and reading scores in three-level models nested in countries (For full model results, see Tables 4, 5). Dashed lines indicate non-significant effects, all other paths are significant at *p* < 0.001

To test Hypothesis 2b on the potential mediating role of a discriminatory climate in the link between minority status and achievement, as well as to test Hypothesis 3 on whether school belonging (Hypothesis 3a) and attitudes toward learning (Hypothesis 3b) further mediate these effects, multilevel mediation models (three levels: students, schools, and countries) were run separately for math (Table 4) and reading performance (Table 5). Ethnic minority status (along with individual-level control variables) was included as a predictor of discriminatory school climate. Then, discriminatory school climate was tested as a predictor of math and reading scores as outcomes directly and indirectly through two additional mediators: school belonging and attitudes toward learning. The associations between discriminatory school climate, school belonging, attitudes toward learning, and test scores were tested at both the individual and school levels (see Fig. 1). The mean and variances of the attitudes toward learning, belonging, and reading/math scores were defined at all three levels in the mediation models. These outcome variables were tested as indicator variables at the individual level and as latent aggregates at higher levels. All the direct effects were kept in the model, and the significance of all indirect effects was tested (Table 6). Note that these are random intercept models (see SOM.2 for alternative random-effects mediation models).

**Table 4:** Multilevel mediation model predicting PISA 2018 math scores

	Discrimination		School belonging		Attitudes to learning		Math	
	*B*	SE	*B*	SE	*B*	SE	*B*	SE
*Ind level*								
SES	−0.06^***^	0.01	0.07^***^	0.00	0.04^***^	0.01	14.04^***^	1.31
Gender	−0.29^***^	0.01	−0.04^**^	0.01	0.15^***^	0.01	−13.31^***^	1.04
Track	−0.16^***^	0.05	0.01	0.01	−0.02	0.02	20.57^***^	6.24
Minority	0.16^***^	0.04	−0.06^*^	0.03	−0.02	0.02	−8.92^***^	2.23
Discrimination			−0.10^***^	−0.10	−0.09^***^	0.00	−11.78^***^	0.63
School Belonging							2.37^***^	0.61
Attitudes to Learning							1.98^***^	0.53
R2	0.03^***^		0.02^***^		0.02^***^		0.10^***^	
(Residual) variance	1.01^***^		0.84^***^		0.94^***^		4999.35^***^	
*School level*								
Ethnic%			0.00	0.00	0.00	0.00	−0.03	0.03
SES%			0.00	0.00	0.00	0.00	−0.55^***^	0.04
Discrimination			−0.15^***^	0.01	−0.07^***^	0.01	−29.81^***^	2.64
School Belonging							55.50^***^	13.38
Attitudes to Learning							−11.25	10.25
R2			0.34^***^		0.07^***^		0.21^***^	
(Residual) variance			0.02^***^		0.02^***^		1193.94^***^	
*Country level*								
Mean			−0.02		−0.05		467.54^***^	
Variance			0.04^***^		0.06^***^		1594.11^***^	

The table presents unstandardized regression coefficients and Standard Errors (SE). Intraclass correlations from a null model with four variables as random intercepts at school and country-level respectively: discrimination 0.06, 0.06; Belonging 0.03, 0.04; Attitudes to Learning 0.02, 0.06; Math: 0.23, 0.28. Discrimination is short for discriminatory school climate

*p < 0.05; **p < 0.01; ***p < 0.001

**Table 5:** Multilevel mediation model predicting PISA 2018 reading scores

	Discrimination		School belonging		Attitudes to learning		Reading	
	*B*	SE	*B*	SE	*B*	SE	*B*	SE
*Ind level*								
SES	−0.06^***^	0.01	0.07^***^	0.00	0.04^***^	0.01	13.85^***^	1.11
Gender	−0.29^***^	0.01	−0.04^**^	0.01	0.15^***^	0.01	15.91^***^	0.82
Track	−0.16^***^	0.05	0.01	0.01	−0.02	0.02	19.93^***^	6.15
Minority	0.17^***^	0.04	−0.06^*^	0.03	−0.02	0.02	−12.53^***^	1.93
Discrimination			−0.10^***^	0.00	−0.09^***^	0.00	−17.03^***^	0.92
School Belonging							2.93^***^	0.81
Attitudes to Learning							2.98^***^	0.53
R2	0.03^***^		0.02^***^		0.02^***^		0.14^***^	
(Residual) variance	1.01^***^		0.84^***^		0.94^***^		5183.44^***^	
*School level*								
Ethnic%			0.00	0.00	0.00	0.00	−0.05	0.04
SES%			0.00	0.00	0.00	0.00	−0.57^***^	0.04
Discrimination			−0.15^***^	0.01	−0.07^***^	0.01	−33.78^***^	2.80
School Belonging							59.86^***^	16.85
Attitudes to Learning							−10.48	11.55
R2			0.21^***^		0.07^***^		0.38^***^	
(Residual) variance			0.02^***^		0.02^***^		1194.35^***^	
*Country level*								
Mean			−0.02		−0.05		452.68^***^	
Variance			0.04^***^		0.06^***^		1152.46^***^	

The table presents unstandardized regression coefficients and Standard Errors (SE). Intraclass correlations from a null model with four variables as random intercepts at school and country level respectively: reading: 26, 0.22; the rest is the same as in Table 4. Discrimination is short for discriminatory school climate

*p < 0.05; **p < 0.01; ***p < 0.001

**Table 6:** Indirect effects for mediation models predicting math and reading scores

	Math			Reading		
Individual Level	*B*	SE	*t*	*B*	SE	*t*
Minority-Discrimination-Achievement	−1.94^***^	0.53	−3.66	−2.82^***^	0.75	−3.74
Discrimination-Belonging-Achievement^a^	−0.24^***^	0.06	−3.86	−0.29^***^	0.08	−3.57
Discrimination-Attitudes to Learning-Achievement^a^	−0.19^***^	0.05	−3.64	−0.28^***^	0.05	−5.53
Minority-Discrimination-Belonging -Achievement	−0.04^*^	0.02	−2.41	−0.05^*^	0.02	−2.43
Minority-Discrimination-Attitudes to Learning -Achievement	−0.03^*^	0.01	−2.36	−0.05^**^	0.02	−2.85
*School Level*						
Discrimination-Belonging-Achievement^a^	−8.09^***^	1.95	−4.16	−8.67^***^	2.50	−3.46
Discrimination-Attitudes to Learning-Achievement^a^	0.83	0.76	1.10	0.78	0.86	0.91

Discrimination is short for discriminatory school climate

*p < 0.05; **p < 0.01; ***p < 0.001

^a^These indirect effects were replicated in separate mediation models for students from ethnic minority and majority groups

All analyses were performed in Mplus 8.3 (Muthén and Muthén, 1998–2017), using maximum likelihood with robust standard errors (MLR) estimation, which is robust to deviations from normality. Missing data were handled using full information maximum likelihood (FIML). FIML uses all available data without imputing missing data, which may introduce randomness in the data. Thus, it is unbiased and preferable to other methods (Dong and Peng, 2013). Survey weights were used throughout the analysis. While the students in the PISA samples were chosen randomly from each country, their selection probabilities varied. Thus, using survey weights ensured the calculation of appropriate estimates of population parameters and their sampling error, and allowed for valid estimates and inferences of the population to be made by considering the complex sample design used to select individual participants for PISA (for further details on weights, see OECD, 2019b). Moreover, since the individual-level predictors were already standardized with respect to the OECD countries, additional centering was not applied to the data. The uncentered models were preferred as they are equivalent to grand-mean-centered models (Paccagnella, 2006), yet easier to interpret (Please see SOM.3). Specifically, in grand-mean-centered models, the school-level effect of discrimination, which was tested at both the individual and school levels, should be interpreted as the difference between its individual and school-level effect (Brincks et al. 2017). In other words, the between-level effect of the discriminatory school climate can be considered a partial effect, when controlling for interindividual differences in student perceptions of the climate at the individual level (Lüdtke et al. 2009). The results remained the same regardless of the centering option (see SOM.3).

All measures, analyses, data exclusions (if any), and additional information about model specifications (see SOM.4) were reported. The raw data and specific research materials are available on the OECD webpage (OECD, n. d.) All the Mplus syntax/outputs/data are available on the Open Science Framework (OSF) page (Baysu et al. 2021). Finally, the word “effect” here was used to refer to directional associations as in regression, which helps differentiate them from bidirectional associations as in correlations. This does not imply causality.

## Results

The correlations in Table 1 show that minority background was associated with stronger perceptions of discriminatory school climate, and these perceptions were significantly associated with lower math and reading scores, more negative attitudes to learning, and lower school belonging both at the individual and school levels.

### Multilevel Moderated Regression

As can be inferred from Tables 2, 3 (*control-only* and *random slopes* models), all the individual-level control variables and students’ minority backgrounds had significant effects on math and reading scores, despite their significant variation across schools and countries: on average, those from higher SES and ethnic majority backgrounds and those attending general school tracks performed better. While boys performed better in math, girls performed better in reading. At the school level, only the SES composition of the school mattered, so those attending schools with higher concentrations of students from low-SES backgrounds performed worse on tests.

In support of Hypothesis 1 (i.e., discriminatory school climate is associated with lower performance across all students), it was found that when students perceived a more discriminatory school climate, they performed worse in math and reading (Tables 2, 3, *main effects* and *random slopes* models), a finding that held for perceived discriminatory school climate operationalized at both the individual and school levels, and after taking into account the significant variation of the effect of discrimination on performance across schools and countries in the random-effects models. In further support of these findings, the descriptive correlations between discriminatory school climate and performance were negative for students from both ethnic minority and majority groups in almost every country, despite varying in size (see Table S1 in SOM.1).

To interpret the effects in the regression models, the estimated achievement scores were calculated for a given value of perceived discrimination based on *random slope* models (see SOM.5 for the calculations). At the individual level, the differences in the predicted math scores between the lowest and highest levels of perceived discrimination were 56 points for students from minority groups and 53 points for those from majority groups. The differences in the predicted reading scores were 79 for students from minority groups and 76 points for those from majority groups. Because 40 PISA points are equivalent to 1 year of schooling (OECD, 2019c), perceived discrimination can make a difference that is equivalent to up to 1 year of schooling for math and 2 years of schooling for reading scores. In addition to calculating the difference in predicted scores at the lowest and highest levels of perceived discrimination, the difference in predicted scores was also calculated at the 20th and 80th percentiles of perceived discrimination. The difference in the predicted scores was then 27 points in math and 38 points in reading for students from minority groups and 26 points in math and 36 points in reading for those from majority groups.

For the moderation hypothesis (Hypothesis 2a; i.e., the effect of a discriminatory school climate on achievement would differ among pupils from the majority and minority groups), a statistically significant interaction between ethnic minority background and discriminatory school climate was found. However, the effect was small, explaining little (<1%) additional variance in performance *(moderation minority* model in Tables 2 and 3). As indicated by the *random slopes* models, when considering the significant variation in the effects of discrimination and minority status across schools and countries, the average random effect of the interaction was not significant, nor was its variation across schools and countries. Thus, the results did not support moderation Hypothesis 2a that the negative association between discriminatory school climate and academic performance would be stronger for students from ethnic minority groups.

### Multilevel Mediated Regression

The *discrimination* mediation models (Tables 4, 5) and the indirect effects (Table 6) show support for Hypothesis 2b (i.e., the mediation hypothesis that adolescents from ethnic minority groups would perceive a more discriminatory climate and in turn perform worse). Accordingly, adolescents from ethnic minority groups experienced a significantly more discriminatory school climate than adolescents from ethnic majority groups, in line with the descriptive mean differences within each country (see Table S1 in SOM.1). In turn, a discriminatory school climate predicted lower math and reading scores. Looking at the indirect effects (Table 6), the negative indirect effects of an ethnic minority background on both math and reading scores via discriminatory school climates were small but significant. Thus, confirming Hypothesis 2b, adolescents from ethnic minority groups experienced more discriminatory climates and, in turn, worse achievement than those from majority groups.

The *school belonging* and *attitudes toward learning* mediation models presented in Tables 4 and 5 show support for Hypothesis 3 (i.e., school belonging and the value attributed to learning would mediate the negative effects of a discriminatory school climate on academic performance). As expected, discriminatory school climate—regardless of whether it was calculated at the individual or school level—predicted lower school belonging and the value attributed to learning. These effects, however, should be interpreted in light of the small ICCs for the mediators at the school level (Tables 4, 5). Higher school belonging and perceiving school as more valuable, in turn, predicted higher math and reading scores; although these effects were again small, they were significant, and the effects of school belonging on test scores were also replicated at the school level. Looking at the indirect effects (see Table 6), the negative indirect effects of a discriminatory school climate on both test scores via school belonging and attitudes toward learning were small but significant, and the school belonging path was also replicated at the school level. In sum, the results provide support for the mediational role of attitudes toward learning and school belonging. Discriminatory school climate predicted lower test scores, both directly and indirectly, via lower school belonging and more negative attitudes toward learning.

### Additional Analyses

Several additional analyses were conducted to test the robustness of the effects of a discriminatory school climate. *First*, the robustness of the mediation model that tested Hypothesis 3 was tested across pupils from the ethnic minority and majority groups. In this model, which was run once on the minority sample and once on the majority sample, discriminatory school climate was a predictor along with individual- and school-level control variables, school belonging and attitudes toward learning were the mediators, and math or reading scores were the outcomes. Negative direct and indirect effects of discriminatory school climate on math and reading scores via lower school belonging and more negative attitudes toward learning were replicated among adolescents from both the ethnic minority and majority groups.

To rule out the possibility that the reported effects of a discriminatory climate—which was measured as pupils’ perceptions of teacher behaviors—have less to do with how schools and school representatives deal with ethnic and racial issues, but merely tap into a lack of perceived teacher support, a series of additional analyses were run to check for the robustness of the findings. Reading scores were the main focus because the PISA data include two additional teacher support variables that are specific to language classes. Whereas a *supportive climate* measured teachers’ general and instrumental support to students during these classes, *perceived teacher support* measured the more personal and affective support that the student perceived during these classes. Therefore, the *supportive climate* was treated as an additional control variable in all analyses reported above, and *perceived teacher support* was treated as an additional mediator along with school belonging and attitudes toward learning for the effects of discriminatory school climate on reading scores.

Accordingly, the second analysis was a multilevel regression analysis with supportive climate as an additional control variable, which replicated the main effects model in Table 3 for reading scores. The negative effects of a discriminatory school climate on reading scores were robust at both the individual and school levels; a supportive climate had a small positive effect at the individual level (*B* = 2.07, SE = 0.44, *p* < 0.001).

Third, a multilevel mediation model was run (depicted in Fig. 1) with supportive climate as an additional control variable, which indicated the robustness of the direct and indirect effects of a discriminatory school climate on reading scores when controlling for the positive effects of a supportive climate on belonging (*B* = 0.12, SE = 0.00, *p* < 0.001), attitudes toward learning (*B* = 0.13, SE = 0.01, *p* < 0.001), and reading scores (*B* = 1.12, SE = 0.42, *p* = 0.007).

Fourth, a mediation model for reading scores was tested, with perceived teacher support as an additional mediator. In this model, discriminatory school climate was a predictor, along with minority status, supportive climate, and other individual- and school-level control variables; school belonging, attitudes toward learning, and perceived teacher support were treated as mediators, and reading score was the outcome. The negative direct and indirect effects of a discriminatory school climate on reading scores via lower school belonging and attitudes toward learning remained robust. Additionally, discriminatory school climate had a negative effect on perceived teacher support at the individual level (*B* = −0.06, SE = 0.00, *p* < 0.001), and school levels (*B* = −0.05, SE = 0.01, *p* < 0.001); teacher support in turn predicted higher reading scores at the individual level (*B* = 5.47, SE = 0.46, *p* < 0.001), but not at the school level (*B* = −8.96, SE = 16.85, *p* = 0.595). The negative indirect effect of a discriminatory school climate on reading scores via lower teacher support was significant at the individual level (*B* = −0.33, SE = 0.04, *p* < 0.001). Importantly, however, these additional effects did not explain away the effects of school discrimination on reading scores via belonging and attitudes toward learning. Details of these additional analyses can be found in SOM.6, and their results as Mplus outputs are stored on the OSF page.

In sum, the above-reported results on the negative effects of discriminatory climates on outcomes are robust across ethnic minority and majority samples and cannot be explained in terms of a lack of teacher support, but are rather related to how teachers, as representatives of the school, are perceived to deal with ethnic and racial issues.

## Discussion

The negative consequences of perceived ethnic discrimination on adolescent adjustment are well documented in the US context (Umaña-Taylor, 2016). Less is known, however, about the consequences of discriminatory *climates* in schools globally—that is, beyond the individual experiences of discrimination and beyond the Western contexts. Addressing these research gaps, this study aimed to examine the association between the perceived discriminatory climate in school and math and reading scores among adolescents from ethnic minority and majority groups across 60 countries, and to investigate potential psychological mechanisms that may account for this link by using the large-scale multilevel and cross-national PISA 2018 dataset. Across 60 countries and adolescents from both ethnic minority and majority groups, the current study found that the more pupils perceived a discriminatory school climate, the lower their scores on the standardized PISA tests for math and reading. Moreover, this negative association could be understood from the fact that discriminatory school climates were associated with a reduced sense of belonging and value attributed to learning and, in turn, with lower test scores. As such, the current research systematically documents the negative association between academic performance and discriminatory school *climates* beyond individual experiences of discrimination. It does so across 60 countries, among which many non-Western ones are included, in contrast to single-country studies that mostly focused on the US (Benner et al. 2018), and for measured standardized test scores for math and reading, in contrast to school grades or GPAs that are difficult to compare across contexts. Below, the three hypotheses are revisited in light of their contributions to the literature.

### Discriminatory School Climates Are Associated with Lower Academic Performance for *All* Pupils

In line with the first hypothesis, both individual and collective perceptions of a discriminatory school climate were associated with lower math and reading scores among adolescents from both ethnic minority and majority groups. This finding extends the growing research on the academic consequences of ethnic discrimination (Benner et al. 2018) and school diversity climate (Thapa et al. 2013) by replicating these negative associations at the individual and school levels across thousands of schools and 60 countries. Despite significant variation across schools and countries, the negative association between perceived discriminatory climate and academic performance was still robust when considering these variations, as in the random slope models. Furthermore, it remained robust after controlling for individual differences, such as whether students went to general or vocational tracks or differences in their SES backgrounds, as well as after controlling for school-level differences in compositions of low SES and ethnic minority groups in school. This latter aspect is important, as it rules out the possibility that school composition factors could explain the lower test scores of pupils.

Overall, the perceived discriminatory climate explained 3–5% of the variance at the individual level and 12–15% of the variance at the school level in math and reading scores, respectively. Although small, these effect sizes are in line with the existing research (see, e.g., Benner et al. 2018’s meta-analysis). To put these effects into perspective, the predicted performance scores at the lowest and highest levels of perceived discrimination were calculated. For instance, at the individual level, the difference in the predicted reading scores between the lowest and highest levels of perceived discrimination was almost equivalent to 2 years of schooling (OECD, 2019c).

It is striking that this negative association between performance and perceived discriminatory climate could be found across *all* pupils, regardless of their ethnic minority or majority status, at the individual and school levels, and across several countries and schools. Of course, due to the cross-sectional nature of this study, causal inferences cannot be made, and it remains possible that those students who performed low on the PISA tests were more likely to perceive a discriminatory school climate. Nevertheless, previous longitudinal research that explored the bidirectionality of the associations between discrimination and academic outcomes via cross-lagged or autoregressive models found that, while earlier perceived discrimination predicted later academic adjustment, the reverse was not true (Cheng et al. 2020). Also, the fact that this study focused on test scores in PISA rather than school outcomes makes it less likely for students to blame their expected/perceived lower performance in PISA on their teachers’ discriminatory beliefs and behaviors in school. In sum, the current findings suggest that when schools are characterized by a discriminatory climate, as is evident from teachers’ misconceptions about the history of some cultural groups or their lower academic expectations for students of some cultural groups, *all* students in that school perform worse academically.

### Students from Ethnic Minority Groups Perceive a More Discriminatory Climate Than Those from Ethnic Majority Groups

This study also scrutinized whether the above-described negative association between perceived discriminatory climate and performance was more consequential for students from ethnic minority groups than for those from ethnic majority groups. In support of this expectation, students from ethnic minority groups perceived higher levels of discriminatory climates at their schools, which replicates previous research (Verkuyten et al. 2019) and supports the mediation hypothesis (2b), which holds that the negative indirect effect of minority status on lower test scores on math and reading was partially accounted for by increased perceptions of discriminatory climate. It is important to note, however, that the strength of the association between discriminatory climate and academic performance was similar for pupils from minority and majority groups. This finding contradicts the moderation hypothesis (2a) and previous research suggesting that the effect of school diversity climate is larger, or is only significant, for students from ethnic minority groups (e.g., Celeste et al. 2019), but is in line with other empirical work highlighting that school diversity climate is beneficial for sense of inclusion (De Leersnyder et al. 2021), belonging, and achievement (Schachner et al. 2019) among students from both ethnic minority and majority groups. Overall, a discriminatory school climate was associated with lower math and reading scores across all pupils, but pupils from ethnic minority groups perceived a more discriminatory climate.

### Discriminatory Climates Are Associated with Lower Belonging and Value Attributed to Learning

Based on the extensive social psychological literature, psychological mechanisms that may potentially explain the associations between discriminatory school climate and academic performance were examined, namely pupils’ sense of school belonging and attitudes toward learning. The mediation analyses showed that when adolescents perceived a discriminatory school climate, they reported lower school belonging and attached less value to learning and effort, which, in turn, was associated with lower performance in math and reading. This finding replicates earlier studies on the mediational roles of belonging and engagement between achievement on the one hand and experiences of discrimination (Baysu et al. 2016), exclusion (Buhs et al. 2006), and school diversity climate (Schachner et al. 2019) on the other. It also adds a novel aspect to the literature, as this provides evidence of the mediational role of attitudes toward learning in the link between discrimination and achievement. In both cases, the direct effects of a discriminatory school climate on math and reading scores were still significant, suggesting only partial mediation and thus calling upon future research to explore other psychological processes that might further explain this link.

Importantly, both psychological processes partially accounted for the link between discriminatory school climate and performance in *both* ethnic minority and majority pupils. This thus suggests that teachers’ negative behaviors toward specific socio-cultural groups are associated with reduced learning of *all* pupils, because they no longer feel like they belong at school and/or do not see the value of learning anymore. This study thus adds to the growing line of research on the negative consequences of experiencing (Benner et al. 2018) as well as witnessing discrimination (Jaurique et al. 2018), which shows that discrimination can jeopardize one’s sense of belonging and engagement (Jaurique et al. 2018) and lead one to devalue learning and trying in school, even when one is not the target of discrimination. However, one can still ask why experiencing or witnessing discrimination, as in experiencing a more discriminatory school climate, is associated with reduced belonging and the value attributed to learning in school. Research suggests that such experiences might threaten one’s sense of identity, whether it is an ethnic (Verkuyten et al. 2019) or organizational identity (Smith et al. 2016), or one’s morality (Jaurique et al. 2018) and fairness concerns (Killen et al. 2013). From a developmental intergroup perspective (Killen et al. 2013), both group- and identity-related as well as fairness-related concerns are salient during adolescence, as adolescents explore group identities as part of their social development, and they develop a deeper understanding of fairness in their moral judgments. Future studies should test these threats and concerns more directly as potential explanations for why discriminatory climates are associated with reduced belonging and the value attributed to learning.

### Strengths

This research made use of the 2018 round of the PISA data. Doing so has three specific advantages that respond to the three current gaps in the literature on discrimination and academic performance. First, in the PISA study, the discriminatory climate in school is measured such that it queries the multicultural or racial school climate. It asks students whether their teachers say negative things or have misconceptions about or lower academic expectations for some cultural groups. Therefore, it measures pupils’ perceptions of the prevalence of stereotypes, prejudice, and discrimination among their teachers as representatives of the school. This operationalization goes beyond the myriad of studies on discrimination that focus on pupils’ own experiences of discrimination and their consequences at the individual level (Benner et al. 2018). Specifically, it acknowledges that these experiences do not happen in a social vacuum, and that the context of school and, more specifically, the school climate of teacher discrimination matter. Furthermore, and in contrast to studies on school diversity climate that focused on various aspects of the diversity-related experiences in school ranging from cultural appreciation (Chang and Le, 2010) to intergroup contact opportunities (Schachner et al. 2019) to combating discrimination (Baysu et al. 2021), this conceptualization allowed for an investigation into the role of discriminatory climate itself, particularly by focusing on perceived discriminatory beliefs and behaviors of teachers. The PISA data also allowed for testing the associations between discriminatory climates both at the individual and school levels across thousands of schools. This goes beyond previous research on school climates, which has often been criticized for relying solely upon individual self-report measures of this climate to predict individual outcomes (Wang and Degol, 2016).

Second, and contrary to most previous work, using the PISA data allowed for the analysis of the associations between discriminatory climate and performance across several national contexts. Almost all educational studies regarding ethnic discrimination (Benner et al. 2018) or school diversity climate (Wang and Degol, 2016) are limited to one specific country or a limited number of countries. Moreover, the literature is heavily weighted toward US samples (Benner et al. 2018), although the topic is internationally relevant, and a cross-national analysis might improve understanding of this issue. This gap was addressed by the current study with the use of the 2018 round of PISA, which includes data across 60 countries and 16,002 schools. This unique cross-national dataset made it possible to test whether the association between the perceived discriminatory climate in school and academic performance could be replicated across countries and schools, something that had not yet been investigated. Third, previous studies mentioned above are limited in that they often rely on self-reported grades or GPA as measures of academic achievement. In contrast to standardized tests, these unstandardized measures make international comparisons difficult, as varying grading systems across schools and countries can be a confounding factor. The PISA data allowed the current study to overcome this issue with its standardized assessments of math and reading across all participants, schools, and countries.

## Limitations

The current study is not without limitations. First, and as discussed above, one should be cautious about the directions of the associations found, given the cross-sectional nature of the PISA data. Second, the data did not have a measure of students’ own experiences of discrimination from teachers, which prevented pitting the effect of a perceived discriminatory climate against that of personal experiences of discrimination. The additional analysis found that those who perceived a more discriminatory climate experienced less teacher support themselves, which was in turn associated with their math and reading scores. Likewise, it could be expected that those who perceived a more discriminatory climate would report more discrimination themselves, but this expectation could not be tested in this dataset. Importantly, however, the additional analyses showed that the negative association between perceived discriminatory climate and performance remained robust after including supportive climate by teachers as a control variable and perceived teacher support as a mediator in the analyses, which suggests that discriminatory climates may predict achievement beyond individual-level experiences, such as teacher support and perhaps also experiences of discrimination. Third, the measure of a discriminatory school climate focuses only on students’ perceptions of teachers’ discriminatory beliefs and behaviors in school. Although teachers are key players in creating a positive diversity climate (Celeste et al. 2021), and previous measures of diversity climate also include items about teachers (Baysu et al. 2021), future studies should also focus on other school-level discriminatory practices, such as admission policies (Bourabain et al. 2020) or specific policies limiting the use of religious symbols or mother tongues in school (Celeste et al. 2019). Finally, due to the available measures in the PISA data, the measure of ethnic minorities in this study refers only to those who are either a language minority or first-/second-generation immigrants. Thus, it excludes those who speak the majority language of their country *and* have a third- or later-generation or no immigration background, like some Black minorities in the UK, or religious minorities, like Alevites in Turkey. However, focusing on first- and second-generation immigrants and language minorities can also be seen as a strength, given that these groups are understudied in the literature on discrimination. In any case, the unique advantages of the PISA data, such as the use of standardized performance measures and testing across several countries and thousands of schools, enhance the ecological validity of the findings reported here—despite being cross-sectional—and thus outweigh its limitations.

## Conclusion

Growing empirical research documents the negative consequences of perceived discrimination on academic outcomes during adolescence. However, this literature is heavily weighted toward US contexts and samples and often focuses on individual experiences of ethnic discrimination among minorities rather than investigating the negative consequences of discriminatory school *climates* for *all* pupils. In addressing these research gaps, the present study established a negative association between perceived discriminatory school climates and academic achievement across 60 countries and adolescents from both ethnic minority and majority groups. Thus, it provides large-scale, multi-country evidence that schools’ ethnic and racial *climates* are associated with the standardized test scores of *all* pupils. To put it simply, when students report that their teachers are prejudiced, hold negative stereotypes, and say negative things about and blame other cultural groups, not only adolescents from ethnic minority groups, but also their peers from ethnic majority groups perform worse in those schools. On the positive side, these findings highlight the long-term benefits of practicing and communicating fairness in helping all students attach a higher value to academic life, feel a higher sense of belonging, and perform well in school. From an applied perspective, these findings suggest that schools can implement specific processes to protect adolescents from these adverse outcomes and promote both the well-being and achievement of *all* students by creating and maintaining a positive school climate that welcomes and respects ethnic-cultural diversity.

## Supplementary information

Supplementary Online Materials
