# Supplementary material for: The Association Between Perceived Discriminatory Climate in School and Student Performance in Math and Reading: A Cross-National Analysis Using PISA 2018
Source: J Youth Adolesc. 2022 Dec 7;52(3):619–36. doi: 10.1007/s10964-022-01712-3 (PMC9884186; doi:10.1007/s10964-022-01712-3)
Supplement: Supplementary file 1 — Supplementary Online Materials [file 10964_2022_1712_MOESM1_ESM.docx]

**Supplementary Online Materials**

**1. Descriptive Associations Between Discriminatory School Climate and Performance in Each Country**

As seen in Table S1, in 53 out of 60 countries, students from ethnic minority groups perceived a significantly more discriminatory school climate than students from ethnic majority groups (with reference to the OECD average). In four countries (Baku, Indonesia, Morocco, Romania), the mean differences were in the opposite direction; in three countries (Tatarstan, Moldova, and Hungary) the mean differences were not significant. When looking at the correlations between discriminatory school climate and math and reading scores, the correlations were almost always significantly negative among students from both ethnic minority and majority groups (except for three countries, Colombia, Korea, and Romania, for math scores among minorities).

Table S1. Separate country statistics for Discriminatory School Climate

|  |  | Discriminatory School Climate (DSC) | | | | | R btw DSC and Math | | R btw DSC and Reading | |
| --- | --- | --- | --- | --- | --- | --- | --- | --- | --- | --- |
|  | Countries | M | SD | N | M min | M maj | Min | Maj | Min | Maj |
| 1 | Albania | -0.10 | 1.16 | 6018 | 0.14 | -0.12 | -0.19 | -0.22 | -0.20 | -0.29 |
| 2 | Argentina | 0.09 | 0.95 | 8189 | 0.09 | 0.08ns | -0.16 | -0.17 | -0.25 | -0.22 |
| 3 | Australia | -0.11 | 0.99 | 10823 | -0.08 | -0.14 | -0.30 | -0.26 | -0.34 | -0.34 |
| 4 | Baku (Azerbaijan) | 0.72 | 1.12 | 3824 | 0.62 | 0.72** | -0.06* | -0.11 | -0.17 | -0.21 |
| 5 | Belarus | -0.13 | 1.02 | 5445 | 0.04 | -0.14 | -0.14 | -0.17 | -0.21 | -0.26 |
| 6 | Bosnia and Herzegovina | 0.21 | 1.07 | 5392 | 0.35 | 0.20 | -0.13 | -0.20 | -0.24 | -0.26 |
| 7 | Brazil | 0.11 | 1.02 | 6851 | 0.58 | 0.08 | -0.14 | -0.26 | -0.24 | -0.32 |
| 8 | Brunei Darussalam | 0.26 | 0.93 | 5100 | 0.29 | 0.03 | -0.32 | -0.22 | -0.37 | -0.25 |
| 9 | Bulgaria | 0.36 | 1.12 | 3819 | 0.40 | 0.35* | -0.04** | -0.13 | -0.17 | -0.23 |
| 10 | Chile | -0.10 | 0.99 | 5338 | 0.03 | -0.12 | -0.32 | -0.25 | -0.38 | -0.35 |
| 11 | Chinese Taipei | 0.17 | 0.95 | 7078 | 0.36 | 0.13 | -0.14 | -0.17 | -0.22 | -0.24 |
| 12 | Colombia | 0.10 | 0.98 | 5625 | 0.58 | 0.09 | -0.01 ns | -0.19 | -0.29 | -0.31 |
| 13 | Costa Rica | -0.27 | 0.91 | 6305 | -0.14 | -0.29 | -0.10 | -0.16 | -0.23 | -0.24 |
| 14 | Croatia | 0.02 | 1.01 | 5689 | 0.14 | 0.01 | -0.27 | -0.23 | -0.31 | -0.31 |
| 15 | Dominican Republic | 0.45 | 1.11 | 1800 | 0.62 | 0.43 | -0.26 | -0.25 | -0.38 | -0.33 |
| 16 | Estonia | 0.02 | 0.96 | 4852 | 0.23 | -0.02 | -0.28 | -0.21 | -0.31 | -0.29 |
| 17 | Germany | -0.04 | 0.94 | 2697 | 0.24 | -0.14 | -0.27 | -0.21 | -0.33 | -0.27 |
| 18 | Greece | 0.34 | 1.00 | 5693 | 0.42 | 0.32 | -0.14 | -0.23 | -0.24 | -0.31 |
| 19 | Hong Kong | -0.03 | 1.01 | 5610 | -0.01 | -0.05 | -0.26 | -0.27 | -0.33 | -0.33 |
| 20 | Hungary | 0.13 | 1.00 | 4406 | 0.16 | 0.13ns | -0.29 | -0.30 | -0.31 | -0.33 |
| 21 | Iceland | -0.26 | 0.99 | 2481 | 0.12 | -0.29 | -0.38 | -0.32 | -0.49 | -0.39 |
| 22 | Indonesia | 0.24 | 1.08 | 11687 | 0.22 | 0.25 | -0.20 | -0.20 | -0.32 | -0.30 |
| 23 | Ireland | -0.30 | 0.87 | 4706 | -0.21 | -0.33 | -0.17 | -0.20 | -0.24 | -0.26 |
| 24 | Italy | -0.12 | 1.00 | 9067 | 0.09 | -0.19 | -0.23 | -0.23 | -0.36 | -0.33 |
| 25 | Jordan | 0.39 | 1.07 | 8177 | 0.43 | 0.37 | -0.14 | -0.09 | -0.22 | -0.18 |
| 26 | Kazakhstan | 0.12 | 1.10 | 16350 | 0.16 | 0.11 | -0.14 | -0.20 | -0.24 | -0.29 |
| 27 | Korea | -0.54 | 0.90 | 6593 | 0.28 | -0.55 | 0.01 ns | -0.18 | -0.12 | -0.23 |
| 28 | Kosovo | 0.22 | 1.00 | 4427 | 0.44 | 0.21 | -0.13 | -0.12 | -0.27 | -0.26 |
| 29 | Latvia | 0.04 | 0.96 | 4607 | 0.14 | 0.02 | -0.23 | -0.22 | -0.31 | -0.31 |
| 30 | Lithuania | 0.15 | 1.04 | 5711 | 0.47 | 0.12 | -0.27 | -0.25 | -0.33 | -0.33 |
| 31 | Macao | -0.14 | 0.84 | 3753 | -0.12 | -0.18* | -0.17 | -0.19 | -0.26 | -0.20 |
| 32 | Malaysia | 0.25 | 0.93 | 5896 | 0.31 | 0.21 | -0.31 | -0.32 | -0.36 | -0.36 |
| 33 | Malta | 0.29 | 1.02 | 2836 | 0.30 | 0.18* | -0.27 | -0.32 | -0.37 | -0.36 |
| 34 | Mexico | 0.09 | 1.00 | 4358 | 0.40 | 0.08 | -0.16 | -0.19 | -0.32 | -0.31 |
| 35 | Moldova | 0.04 | 0.88 | 4956 | 0.02 | 0.03ns | -0.08 | -0.12 | -0.17 | -0.21 |
| 36 | Montenegro | 0.15 | 1.15 | 5616 | 0.37 | 0.12 | -0.12 | -0.11 | -0.28 | -0.25 |
| 37 | Morocco | 0.59 | 0.96 | 2959 | 0.56 | 0.85 | -0.18 | -0.17 | -0.19 | -0.15 |
| 38 | Moscow Region | 0.17 | 1.09 | 1768 | 0.30 | 0.15 | -0.22 | -0.21 | -0.28 | -0.27 |
| 39 | New Zealand | -0.01 | 0.95 | 5195 | 0.06 | -0.03 | -0.26 | -0.22 | -0.31 | -0.29 |
| 40 | North Macedonia | 0.14 | 1.01 | 5202 | 0.35 | 0.11 | -0.18 | -0.19 | -0.26 | -0.28 |
| 41 | Panama | 0.29 | 1.03 | 2147 | 0.41 | 0.25 | -0.23 | -0.19 | -0.27 | -0.27 |
| 42 | Peru | 0.04 | 0.87 | 2664 | 0.24 | 0.03 | -0.34 | -0.20 | -0.48 | -0.29 |
| 43 | Philippines | 0.59 | 0.94 | 6588 | 0.60 | 0.55 | -0.27 | -0.31 | -0.33 | -0.37 |
| 44 | Poland | 0.09 | 0.97 | 5173 | 0.52 | 0.08 | -0.45 | -0.20 | -0.47 | -0.27 |
| 45 | Portugal | -0.19 | 0.94 | 5057 | -0.01 | -0.21 | -0.25 | -0.26 | -0.30 | -0.34 |
| 46 | Romania | 0.08 | 0.91 | 4606 | 0.03 | 0.07* | 0.03* | -0.19 | -0.03 | -0.29 |
| 47 | Russian Federation | 0.08 | 1.08 | 6553 | 0.15 | 0.07 | -0.17 | -0.20 | -0.22 | -0.28 |
| 48 | Saudi Arabia | 0.60 | 0.98 | 5473 | 0.62 | 0.58 | -0.08 | -0.09 | -0.15 | -0.13 |
| 49 | Serbia | 0.13 | 1.09 | 4970 | 0.16 | 0.11** | -0.13 | -0.14 | -0.20 | -0.23 |
| 50 | Slovak Republic | 0.31 | 0.97 | 4975 | 0.55 | 0.28 | -0.13 | -0.24 | -0.27 | -0.32 |
| 51 | Slovenia | 0.25 | 0.96 | 5262 | 0.48 | 0.22 | -0.19 | -0.29 | -0.21 | -0.38 |
| 52 | Spain | -0.11 | 0.99 | 28142 | 0.00 | -0.15 | -0.23 | -0.20 | -0.34 | -0.31 |
| 53 | Switzerland | 0.01 | 1.03 | 3869 | 0.16 | -0.08 | -0.28 | -0.29 | -0.40 | -0.35 |
| 54 | Tatarstan (RUS) | 0.13 | 1.08 | 5202 | 0.15 | 0.13ns | -0.20 | -0.23 | -0.25 | -0.30 |
| 55 | Thailand | 0.46 | 1.00 | 8390 | 0.60 | 0.45 | -0.17 | -0.25 | -0.25 | -0.32 |
| 56 | Turkey | 0.36 | 1.03 | 6673 | 0.54 | 0.34 | -0.03 | -0.14 | -0.15 | -0.20 |
| 57 | Ukraine | -0.02 | 0.95 | 5297 | 0.05 | -0.07 | -0.24 | -0.19 | -0.31 | -0.25 |
| 58 | United Kingdom | -0.29 | 0.92 | 2504 | -0.22 | -0.31 | -0.23 | -0.22 | -0.27 | -0.34 |
| 59 | Uruguay | 0.05 | 0.99 | 3178 | 0.28 | 0.02 | -0.24 | -0.19 | -0.26 | -0.27 |
| 60 | Vietnam | -0.31 | 0.88 | 5340 | 0.02 | -0.33 |  |  |  |  |
| *Note.* All mean differences (t-test) and all correlations (Pearson R) are significant at *p* < .001 unless otherwise indicated; ns indicates non-significant differences/correlations | | | | | | | | | | |

**p < .05 **p <.01*

**2. Random Slopes in Mediation Models**

As a robustness check, to ensure that the effects would be similar taking into account potential variation across schools and countries, a simplified mediation model was tested by removing any effects on discrimination. In this mediation model, ethnic minority status and discriminatory school climate were the predictors, school belonging, and attitudes towards learning were the mediators, and math or reading scores were the outcomes. In total, six random slopes that were of theoretical interest were defined: The effects of individual-level minority status and discriminatory climate on the mediators and performance. This model also included the fixed effects of the individual-and school-level control variables on both mediators and outcomes as well as the school-level mediation between aggregate discriminatory climate, both mediators, and performance, as in other models. Results showed that five out of six random slopes significantly varied across schools and countries (except for the effect of minority status on belonging which only varied across countries). Average random effects of minority status and discrimination on the mediators and outcomes were very similar to those fixed effects reported in the paper, besides the fixed effects of all other variables.

This study had to test a simplified model because testing random slopes in the main mediation models reported in the paper was not possible. Technically simultaneous estimation of several random slopes (including those of control variables) increases the complexity of the models and is time-consuming. Moreover, Mplus does not allow for missing values in covariates in random slopes. It is possible to model the random effects of predictor variables with missing values (by not modeling their means and variances) but not possible for those of the mediator variables like discrimination. Thus, estimating the random effect of the minority status on discrimination and that of discrimination on additional mediators and outcomes in the same analysis was not possible. Moreover, as these models produced more parameters than the clusters (due to the addition of random effects), yielding error messages, all additional parameters were removed from the school level (the means and variances of discriminatory school climate and of the percentages of ethnic minority and low SES students and the covariance between the two mediators belonging and attitudes towards learning at the school level) in line with the model specifications for the additional analyses described in SOM.6. Model outputs are stored in the OSF.

**3. Centering**

The effects in Table 2 and 3 *Moderation Minority* and *Random Slopes* models were replicated using grand mean centering of all the predictor variables, and the results remained the same. These models were also replicated using group mean centering for the only variable that was tested at both individual and school levels, i.e., discriminatory school climate. While the effects of discriminatory school climates at the school level were larger in these models, the results remained the same. The results of these additional analyses can be requested from the corresponding author.

**4. Model Specifications for the Main Analyses**

Mplus handles missing values on mediators and outcomes automatically. For predictors, adding/estimating means and variances allow for FIML. The mean and variance parameters for the continuous predictor variables (discriminatory school climate at individual or school levels, SES at the individual level, the percentages of ethnic minority and low SES students at the school level) were estimated in most models. There were a few exceptions to estimating missing values. First, in random slope models, these parameters were not estimated at the individual level (i.e., means and variances of SES and discriminatory school climate). Because random slope models also allowed variation of individual-level SES and discriminatory school climate across both school and country levels, estimating their missing values at the individual level led to modeling issues. Second, in the interaction models, when the interaction is defined within the Mplus syntax, as was done here, Mplus does not estimate the missing values for the interaction. Third, the means and variances for dummy-coded predictors (minority status, gender, and school track) were not estimated as these yielded error messages. However, results were robust regardless of whether these additional parameters were estimated, and thus missingness, for dummy-coded predictors. Only the covariances between mediators at individual and school levels were modeled, whereas the covariances between predictors were not modeled in any of the models due to the added complexity of the analyses.

In estimating the intra-class correlation for reading scores, the model gave an error message regarding the saddle point, but this message can be ignored as the analysis produced all the necessary standard errors in accordance with the advice here (<https://www.statmodel.com/download/Saddle%20point.pdf>)

**5. Predicted Values**

Based on the *Random slopes* models that took into account the interaction, one can calculate the predicted achievement scores as intercept + discrimination*value + minority*value + interaction*value. Predicted scores were first calculated for those at the lowest (-1.16) and highest values of discrimination (3.18), and then at the 20^th^ (-1.16) and 80^th^ percentiles (0.94). For instance, for math at the lowest level discrimination for the minority, the calculation would be as follows: 468.59+ (-12.23 *-1.16) + (-9.44*1) + (-0.61*-1.16). Accordingly, for math performance, the calculated values at the lowest and highest levels of discrimination would be 473.97-418.29 for the minority and 482.71-429.67 for the majority. The estimated math scores at the 20^th^ and 80^th^ percentiles would be 473.97-447.09 for the minority, 482.71-457.10 for the majority. For reading performance, the calculated values at the lowest and highest levels of discrimination would be 458.71-380.09 for the minority and 472.00- 396.41 for the majority. The estimated math scores at the 20^th^ and 80^th^ percentile would be 458.71-420.76 for the minority, and 472.00-435.51 for the majority. The difference scores reported in the paper were calculated by subtracting the performance scores at the low and high levels of discrimination (e.g., 473.97-418.29= 55.67). These calculations are based on average random effects.

**6. Model Specifications for the Additional Analyses**

In the *first* additional analysis whereby the mediation model was tested separately for the minority math scores, adding the covariance between the two mediators at the school level caused an error message, so it was dropped. Results were robust with or without the added covariance.

In the *third and fourth* additional analyses, supportive climate was tested as an additional control variable in the mediation model, and in the fourth analysis, perceived teacher support as an additional mediator. These models produced more parameters than the clusters, which yielded error messages. Thus, all additional parameters were removed from the school level (the means and variances of discriminatory school climate, and of the percentages of ethnic minority and low SES students; the covariance between the two mediators belonging and attitudes towards learning at the school level) from both models. From the fourth model, the attitudes towards learning mediation path was also removed from the school level, in addition to the covariances between mediators at the individual level (between emotional support, belonging, and attitudes towards learning). Removing these parameters reduced the number of parameters (< clusters) and the complexity of the models. The results were robust regardless of estimating these parameters. The results from the simpler models are reported in the paper, whereas both simpler and more complex model outputs are stored on the OSF page.

**7. Additional Information on PISA Study: Scale Development and Sampling**

In PISA 2018, as in previous cycles, questionnaires were developed by the Questionnaire Expert Group (QEG) through regular meetings where the experts revised questionnaire drafts as well as feedback from countries. Specific standards regarding quality assurance, such as a review by the countries/economies and linguistic translatability assessments are explained in Chapter 2 of the PISA 2018 Technical Report (OECD, 2019b). Moreover, to ensure that the measures of student background, practices, attitudes, and perceptions are comparable across countries, construct validation was achieved by checking international consistencies within and across countries as well as testing cross-cultural validity (via testing invariance of item parameters). These strategies are explained in detail in Chapter 16 of the Technical Report. Chapter 16 also describes how each variable is derived and calculated.

In all the countries (except for Russia), a two-stage stratified sample design was followed. The first-stage sampling units consisted of individual schools having 15-year-old students. Schools were sampled systematically from a comprehensive national list of all PISA-eligible schools in accordance with the school size (called a systematic probability proportional to size sampling). Schools in the sampling frame were stratified according to different criteria, such as school type and region. A minimum of 150 schools were selected in each country (or all schools if the country had fewer than 150 schools). The second-stage sampling unit consisted of a complete list of each sampled school’s 15-year-old students. Students were selected with equal probability from this list, to reach the target size of 42 students per school (the minimum allowed target size was 25 students). The overall target was a minimum sample size of 6300 students per country (or 5250 depending on the survey options). The details are described in Chapter 4 of the PISA 2018 Technical Report (OECD, 2019b). Looking at the actual results from the current analyses (the null model), the average cluster size was 27.67 at the school level (i.e., around 28 students per school) and 7460.288 at the country level (around 7460 students per country)

Schools are compensated for their participation in PISA while the amount varies across countries (Engel & Rutkowski, 2020).

Engel, L. C., & Rutkowski, D. (2020). Pay to play: What does PISA participation cost in the US? Discourse: *Studies in the Cultural Politics of Education*, 41(3), 484-496.

**8. Moderation of the Effects of Discriminatory School Climates by School Belonging and Attitudes Towards Learning**

Instead of the mediation analysis, the alternative moderation hypothesis, i.e., that the negative effects of discriminatory school climates could be different for students that differ in their school belonging levels or their attitudes toward learning was tested. For simplicity, these were random intercept models. Not surprisingly in such a big sample, the interactions between individual-level discrimination and belonging (on Math, B = 1.37, SE = .19, p <.001; on reading B = 1.77, SE =.22, p <.001) as well as discrimination and value attributed to learning (on Math, B = 0.77, SE =.22, p <.001; on reading B = 0.99, SE = .22, p <.001) were significant. As the simple slope analyses indicate (Figures S1 and S2), discrimination had negative effects on math achievement both among those who perceived lower belonging (B = -13.20, SE = .66, t = -20.03, p <.001) and among those who perceived higher belonging (B = -10.54, SE = .66, t = -15.92, p <.001); as well as those who attributed less value to learning (B = -12.68, SE = .59, t = -21.40, p <.001) and those who attributed more value to learning (B = -11.12, SE =.71, t = -15.70, p <.001). The results on reading scores were very similar.

Figure S1. Discriminatory climate effects on math scores moderated by school belonging

Figure S2. Discriminatory climate effects on math scores moderated by attitudes towards learning
